# Effect of stage-matched educational intervention on use of institutional delivery in Northwest Ethiopia: using community readiness model

**DOI:** 10.11604/pamj.2024.48.16.37504

**Published:** 2024-05-20

**Authors:** Adane Nigusie, Telake Azale, Mezgebu Yitayal, Lemma Derseh

**Affiliations:** 1 Department of Health Promotion and Health Behavior, Institute of Public Health, College of Medicine and Health Sciences, University of Gondar, Gondar, Ethiopia,; 2 Department of Health Systems and Policy, Institute of Public Health, College of Medicine and Health Sciences, University of Gondar, Gondar, Ethiopia,; 3 Department of Epidemiology and Biostatics, Institute of Public Health, College of Medicine and Health Sciences, University of Gondar, Gondar, Ethiopia

**Keywords:** Community readiness assessment, education, effect, health promotion, non-randomize

## Abstract

**Introduction:**

even though there are many initiatives to improve institutional delivery, there are low service utilization and community readiness for institutional delivery in Ethiopia. This study assessed the role of community readiness on delivery service use.

**Methods:**

a pre-and post-test design with a control group was used for the evaluation of the stage-matched educational intervention following the protocol of the community readiness assessment model. Based on the baseline assessment of community readiness among 15 kebeles where the study was conducted, the overall score of nine kebeles was below stage-5 out of the nine stages, which were targeted for the intervention. The intervention group (n= three kebeles) participated in the stage-matched intervention for 15 months, while the control group (n= three kebeles) were not given the intervention. The data were analyzed using the difference in difference (DiD) method.

**Results:**

there were significant improvements in a stage of change for the promotion of institutional delivery (p-value <0.001) and institutional delivery use (p-value <0.001) in the intervention group as compared to the control group. The study revealed that the intervention influenced community resource allocation (at marginally significant levels), improved leader-ship quality of prevention, and community climate to supportive prevention efforts. There was evidence that the intervention (health promotion) also increased service use at a significant level.

**Conclusion:**

the community readiness-based intervention (health promotion) can be useful to measure the combined attitude and behavior towards institutional delivery services. The village-based mobilizer approach had a positive effect on institutional delivery use and the level of community readiness on the promotion of institutional delivery.

## Introduction

Communities vary greatly in their interest and willingness to try new prevention strategies and to be involved as a change agent [1,2]. Given the evidence for the prevalence of specific health issues and the level of community readiness for the promotion of the specified health issues, it is vital to design appropriate behavioral change interventions [3,4]. In sub-Saharan Africa, the prevalence of institutional delivery has been progressing in a steady manner and impacts on maternal and child health [5]. In Ethiopia, institutional delivery service use and the community´s level of readiness for the promotion of institutional delivery service use are low [6,7]. Community-based intervention using a behavior change model is a challenging activity for researchers [3]. The interventions based at community level were effective for the improvement of health service utilization in the rural communities [8]. Scrutinizing community level behavior change is essential and appropriate for objectives based at the community level. Community level outcomes, including community involvement, increases in social capital, shifts in community norms, and readiness for change, are complex and difficult to operationalize. Therefore, a comprehensive model that enables a rigorous examination of community-level factors and tests hypotheses about the behavior change of the community is needed. The community readiness model (CRM) provides a comprehensive framework for understanding key community-level factors and developing appropriate strategies to promote the health issue through community support. The CRM framework states that the preparedness of the community to address the specified health issue can be analyzed by both dimension and overall level of readiness, and the factors can easily be characterized and operationalized, which enables quantification for ease of use in evaluation [9].

This study designed an intervention approach of village-based mobilizers through an initiative of “mothers and the week”. Workshops were conducted in the intervention communities and were combined with the “mothers and the week” workshop that introduced the “mothers and the week” component of our study. Participants were trained about the details of the activities of the “mothers and the week” and how to use different promotional items provided by the study teams based on the stage-or dimension-related needs. Using the village based mobilizer for “mothers and the week” initiative as an important caretaker in community-based health promotion [10] existing local structures were invited and used for the intervention; and encouraged to partner with issue leaders at the village level to bring greater public attention to the promotion of institutional delivery use issues. The potential of village based mobilizer for “mothers and the week” to increase issue awareness through agenda-setting has been well established [11]. Further, stage-matched educational intervention has the potential to reinforce individual behavior change through its effects on public attitudes [12]. Therefore, it was our belief that community-level stage-matched educational intervention (health promotion) efforts would reinforce the community to take over the activity aimed at influencing community attitudes and behavior. Moreover, we expected that these community-based stage-matched educational intervention (health promotion) efforts would impact the community's level of readiness and institutional delivery use.

## Methods

**Study setting:** this study was conducted in rural areas of the Central Gondar Zone in 2019 [7].

**Sample and design:** key informant interviews following the protocol of the community readiness model were conducted with community members of low level of readiness in the baseline assessment in 6 Central Gondar zone kebele (3 treatment, 3 control), in a group non-randomized trial of a community-based stage matched educational intervention, prior to and following the intervention work. Baseline and follow-up interviews were 15 months apart. The characteristics and the required criteria of the key informant were briefly described in the baseline assessment for this study [7]. Participants in the stage-matched educational intervention (health promotion) were recruited separately, and interviewees did not participate in the intervention itself. During the baseline assessment, 96 interviews were conducted (on average, six in each kebele). Based on the baseline assessment, the intervention was given for those communities with a low level of readiness; six kebele (3 interventions and 3 controls) with 40 interviews (on average seven in each kebele), all were from the baseline. When post-project assessments were conducted 15-months later, 40 interviews were conducted. Efforts were made to contact the same people as per the suggestion of the CRM; all of the informants interviewed at baseline were re-interviewed. The mean age was 32 years; 42.5% were male and 57.5% were female, and 42.5% were actively involved in the health system.

**Figure 1:**
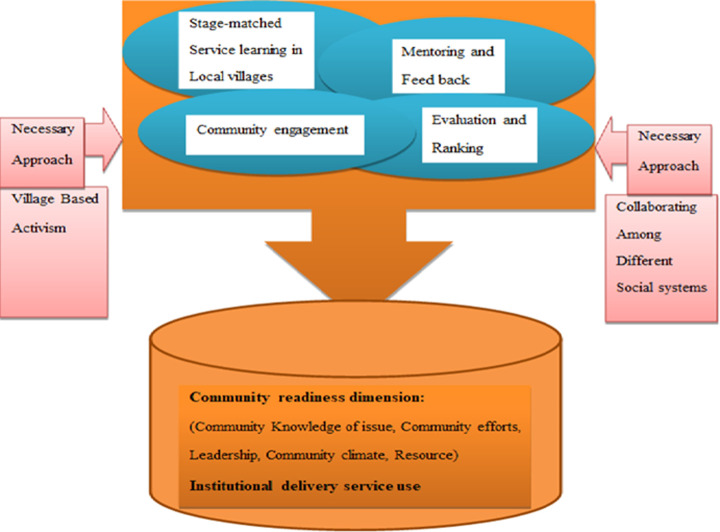
mothers and the week implementation framework

**Development of the stage matched intervention (SMI):** the CRM was used to guide the SMI design, named as mothers and the week. The content of the SMI was prepared based on the baseline proposed activity [7] and remarks in the literature [13-19]. An expert review and a panel discussion were conducted with [7] experts for content validity. The expert group consisted of three health education and behavioral science lecturers who are knowledgeable about the CRM-behavioral intervention, one maternal and child health expert from the district health office, one reproductive health expert, one assistant professor of public health who is knowledgeable on the CRM-behavioral intervention, one implementation research expert, and one midwife who is knowledgeable about maternal health services. The experts were asked to review and suggest 13 components of the SMI for content validity: i) introduction; ii) preliminary findings; iii) community level of readiness to address home delivery (to promote institutional delivery service), iv) agenda of the behavioral change activity(mothers and the week); v) necessary approaches and theoretical frameworks, vi) interconnected strategies, (vii) stage matched service learning in local villages; viii) mentoring and feedback; ix) evaluation and ranking; x) community engagement; xi) behavior change mobilizer roles and activities; xii) educational strategy, and xiii) reporting system, format and content. They were asked to suggest the importance of each component and to incorporate their suggestions. Finally, the SMI was revised by using the results of the experts´ suggestions and opinions, and then completed by the researchers. The final version of the SMI protocol is briefly summarized in (Figure 1). The CRM conveyed stage-specific proposed activities to increase readiness to the next stage that were intended to keep the community on track to address the health problem in a more culturally acceptable way and improve institutional delivery. Based on the proposed activities in line with the score of each dimension described in the baseline assessment [7] training manual was prepared.

**Participants and setting:** one behavior change mobilizer was recruited from each village in the intervention group. We have 48 villages in the intervention group. So, forty-eight participants were recruited for the SMI. The study had ethical approval by the Institutional Review Board of the University of Gondar (Protocol R.No: -O/V/P/RCS/05/1048/2019; Date: 04 March 2019). Participants were asked to mobilize the community for institutional delivery and link it with the health system, and have regular meetings at the village level, weekly with health extension workers, monthly and quarterly with the principal investigator at the kebele level. Based on the performance of institutional delivery use, Antenatal Care, postnatal care, and resource allocation, the principal investigator evaluated and ranked each village; each village would be acknowledged quarterly and a recognition platform was also established.

**The stage matched intervention protocol:** the SMI was designed for village based behavior change mobilizers in the community at the village level to promote institutional delivery service use/prevent home delivery. The 15-month SMI consisted of three components: a) the stage-matched behavior change education strategies based on the CRM level of readiness; b) service use behavior training at each village based on the current participant performance of ANC use, institutional delivery use and PNC; and c) monitoring, evaluation and strong feedback with ranking.

**Figure 2:**
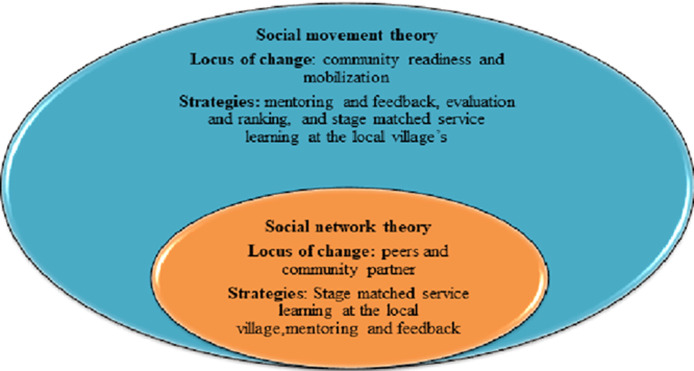
mothers and the week two theoretical frameworks, mothers and the week draw on each theory for specific strategies and locus of change

**Necessary approaches and theoretical frameworks:** the stage matched intervention program was designed for the parts of the community with low stages of readiness at baseline, and the starting point of mothers and the week focused on creating a sense of ownership via strong promotional activities and building trust, which formed the essential set-up for village-based advocacy and collaboration among different social systems [7]. This essential set-up was used for the implementation of tactics, which are designed to target dimensions of the community with a low stage of readiness within four interconnected basic strategies: i) stage-matched service learning in local villages; ii) mentoring and feedback; ii) evaluation and ranking, and v) community engagement (Figure 1). The mothers and the week initiative was built on the assimilation of social network theory [20] and social movement theory [21] (Figure 2). For the appropriate utilization of stage-matched service learning at local villages, and mentoring and feedback strategies to spread messages through village based activist we have used social network theory i.e. peer and community partner networks in collaboration with the existing social systems. According to the social movement theory, we have used the community engagement linked with mentoring and feedback, evaluation and ranking, and stage-matched service learning at the local village´s strategies to align the demand for institutional delivery (i.e., improving the norms of institutional delivery use).

Throughout the initiative of the program, partnerships among different social systems (community gatherings) like Idir, Ekub, and other social events were developed. One kebele consists of an average of 10 villages. From each village, one village activist was recruited for about 15 months. Mothers and the week´s mentoring and feedback strategies included health facility partners, such as a health center-run public relations firm. Evaluation and ranking of the performance helps to guide the village-based activist approach with designing mothers and the week´s promotion platforms and materials. Village-based activists were the heart of mothers and the week; they provided important links to the local community with the health facility. A total of 48 village based activists were recruited and trained from each intervention kebele for the follow-up study. Following the CRM protocol we have prepared a stage-matched intervention material which was community-relevant and culturally appropriate frameworks and messaging strategies. In order to increase community readiness within the 4 interconnected basic strategies, the intervention (implementation of tactics) were given for the selected cohort of communities with low level of readiness at baseline.

**Interconnected strategies:** this strategy targeted the lowest level of the dimensions of community readiness (CR) for the promotion of institutional delivery service utilization at the baseline assessment. The strategy includes stage-matched service learning in local villages, mentoring and feedback, evaluation and ranking, and community engagement.

**Stage-matched service learning in local villages:** mothers and the week were relevant to the community and served as a platform to initiate communication and raise awareness around institutional delivery service use. The primary focus of this strategy was targeted on the lowest score of CRM dimensions at baseline; leadership, community climate and resource allocation for the promotion of institutional delivery service utilization. The village activists, with the assistance of health extension workers, researchers, supervisors, and health center staff, were engaged with the village leaders to advocate and promote institutional delivery service use for the interventions in the village setting that required minimal resources and were responsive to do. The village activists conducted audits of their village´s number of pregnant women; Antenatal Care (ANC) service utilization trends, place of delivery, to address the identified concern they are going to develop strategic plans, and have a regular meeting at the village and kebele level per week and per month respectively with the health extension workers, supervisors, kebele leaders, researchers and cluster health center representatives to discuss the potential activities they perform. From 2019 through 2021, village-based activists were assigned to conduct mobilization activities in the intervention kebele for the promotion of institutional delivery service utilization. We distributed promotional materials on maternal and child health including ANC, institutional delivery service utilization, feeding practices, and immunization services at the village level and at different community gatherings to increase awareness of maternal health service utilization and promotion of institutional delivery service use.

**Mentoring and feedback:** appropriate identification of the skills and knowledge needed for the successful career of the mentee/village based activist, frequent feedback is a core component of the intervention activity. Mentoring the assigned mobilizer (village based activist) needs to have a feedback which allows to acknowledge their strengths and to motivate them to work on the areas of weaknesses. The mobilizer wants the feedback from the supervisor to move forward in their performance/career. If feedback had been provided on a regular and timely basis, the mobilizer would not get bogged down pursuing the wrong path in his/her activity. This strategy is focused on the routine follow-up of the activity given to the village activists and showing their performance on the specified issue via frequent and timely feedback (written, oral, and phone) i.e., the specified issue includes discussions with the community at the village level, ANC follow-up, delivery service and postnatal care (PNC).

**Evaluation and ranking:** mothers and the week program were using a periodical evaluation of the performance and ranking of each village activist based on the plan they had been given. The evaluation was taken at the kebele level every three months, and their overall rank was also announced and recognized with some incentives for better performing village activists. This strategy enables the activists to have a sense of computation and so that they can be involved strongly in being a winner. As a result, they will go for better mobilization and awareness creation for the large community by using the different social systems at the local level with low cost but better performance, which ensures the sustainability of the service utilization since the community considers it as a norm.

**Community engagement:** this strategy was focused on the community knowledge of mothers and the week efforts and resource allocation for the prevention of home delivery activity. Mothers and the week program use the activities on the ground to spread awareness in the large community about the efforts of the initiative. Analysis of the major community stakeholders and building strong relationships were the primary steps taken for this strategy. We have tried to attend different community gatherings to update the attendees/participants on the activities and to link with other administrations. In addition, the village based activists attended many major community gatherings (like Idier, events and coffee ceremony) and many different kebele level coalition meetings to personally engage with corresponding community members from the inception of the program 2019 to the end of the program 2021.

**Tool or instrument:** the tool used for this study was briefly described in the baseline assessment as it was adapted from the community readiness assessment model, which is a semi-structured interview based on open-ended questions relative to each of the five dimensions of readiness [7]. The CRM had two phases, i.e., the assessment phase and the application phase. The assessment phase had four steps: 1) identifying the issue; ii) defining the community with regard to the issue; iii) conducting interviews with key respondents; and iv) scoring the responses of the interviewees. The second phase of community readiness is the application phase, and it has the last two steps: v) developing community-specific strategies to support the stage of readiness as determined within the assessment phase; and x) implementing those strategies. This article provided results on the second two steps based on the baseline assessment score after a stage-matched educational intervention was conducted for 15 months in kebeles with low level of readiness.

**Procedures:** the key informant´s interview was conducted by the principal investigators using the Community Readiness Assessment interview. The duration of the interview was 35 to 60 minutes. The confidentiality of the interview response was assured to the interviewees. The transcription of the responses was simultaneously transcribed in full by the principal investigators. The coding of the transcription was independently coded by two trained coders who were not participating in community readiness ratings. To minimize possible coding errors, the two coders met to reconcile any discrepancies [22].

**Measurement:** demographic variables including sex, educational status, age, responsibility, and stay duration of the key informants were assessed by the principal investigators upon entry into the study. Levels of readiness were assessed using the community readiness model working protocol [23]; the questions were adapted from the CRM to decide the community level of readiness for every subsequent dimension: i) knowledge of issue; ii) community efforts and knowledge on the efforts; iii) leadership; iv) community attitude; and v) resources. Five readiness scores were given for each of the completed interviews, one for every dimension, using dimension-specific rating scales. The given scores were then averaged by each dimension across all key informants to run a summary of dimension readiness scores for the community. Each dimension score corresponded to a stage of readiness, together with an overall average readiness score that was calculated from the five dimension scores. Two trained CRM scorers were involved in the scoring process of the finding for the entire interview and there were no significant deviances to the scoring protocol [7]. Institutional delivery was assessed categorically; “Yes” if women reported they gave their most recent birth (within the last year) at a health institution, and “No” if otherwise.

**Data analysis:** five readiness scores were given for each of the completed interviews, one for every dimension, using dimension-specific rating scales. The given scores were then averaged by each dimension across all key informants to run a summary of dimension readiness scores for the community. Each dimension score corresponded to a stage of readiness, together with an overall average readiness score that was calculated from the five dimension scores. Two trained CRM scorers were involved in the scoring process of the finding for the entire interview and there were no significant deviances to the scoring protocol. Finally, the group non-randomized trial results were analyzed using STATA version 14 for the level of readiness and institutional delivery use for both groups was analyzed using the Diff. command. STATA version 14 was used to analyze the data. Descriptive statistics were used to characterize the key informants and variables of the study. Difference-in-difference was used to analyze differences between pre-intervention and post-intervention values.

**Ethics approval and consent to participate:** we obtained ethical approval for the study from the institutionalized review board, at the University of Gondar (Protocol R.No: -O/V/P/RCS/05/1048/2019; Date: -04 March 2019). An official letter that explains the objectives of the study was written to the respected administration and the zonal health office. The zonal administration and zonal health office successively wrote letters to districts for cooperation, respectively. The objectives and the benefits of the study were explained for the study subjects. Informed written consent was obtained from each participant. The right of the participants to withdraw from the study whenever they wanted to do so was respected. The anonymity and confidentiality of the respondents were ensured.

## Results

**Characteristics of the participants:** forty key respondents completed CRM questionnaires on the issues of promotion of institutional delivery use/prevention of home delivery. In this study, 23 (57.5%) of the respondents were females, and the age of the respondents ranged from 18 to 34 years old, and the majority of the respondents were between the ages of 25 and 34 years. These participants were chosen to be key informants based on their various areas of experience [7]. A minimum of six key informants were interviewed from each kebele. All respondents were used in the scoring process of the level of readiness. The key informants represented varied positions in the kebeles; the most common were different association heads in the kebeles (40%), Health development army leaders (25%), kebele administrators (17.5%), and health extension workers (17.5%) (Table 1).

**Table 1:** socio-demographic characteristics of key respondents at Central Gondar Zone, Northwest Ethiopia, 2019 (n=40)

S.N	Characteristics		Frequency (%)
1	Sex	Male	17 (42.5%)
		Female	23 (57.5%)
2	Educational status	Can't read and write	7 (17.5%)
		Can read and write	6 (15%)
		Primary (grade 1-8^th^)	19 (47.5%)
		Secondary and above	8 (20%)
3	Age	18-24 years	18(45%)
		25-34 years	22 (55%)
4	Responsibility	Kebele administrators	6 (15%)
		Different associations	16 (40%)
		Health extension worker	6 (15%)
		Health development army leaders	12 (30%)
5	How long lived in the kebele	<=5 years	5 (12.5%)
		>5 years	35 (87.5%)

**Figure 3:**
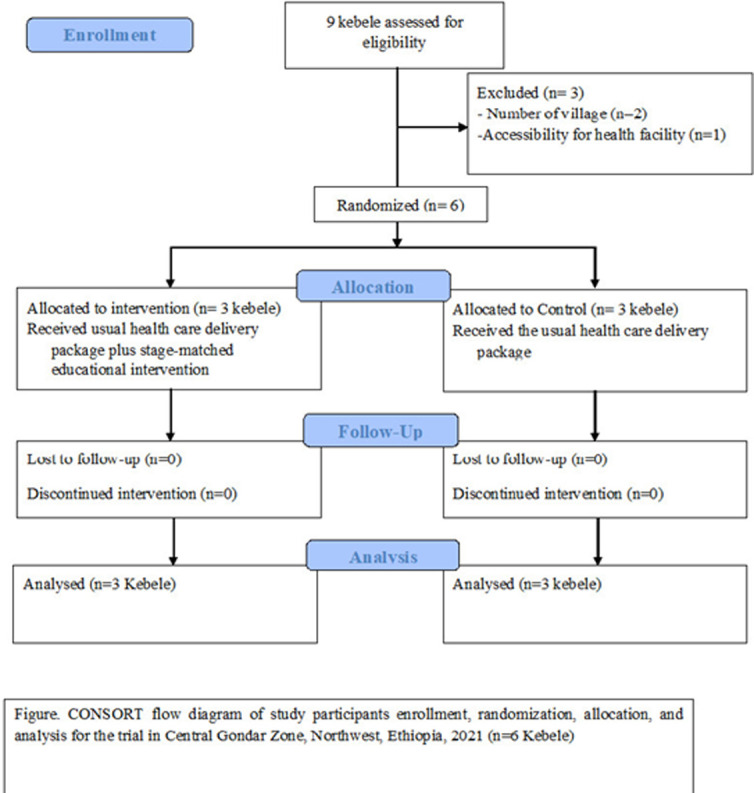
consort flow diagram of study participants' enrollment, randomization, allocation, and analysis for the trial in Central Gondar Zone, Northwest, Ethiopia, 2021 (n=6 Kebele)

**Figure 4:**
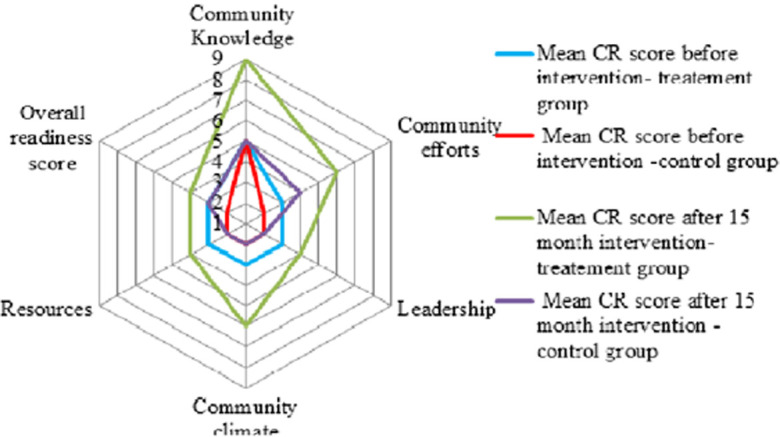
dimension readiness score out of nine point of scale for treatment and control group in Central Gondar Zone, Northwest Ethiopia, 2021

**Community readiness for the promotion of institutional delivery:** the overall levels of community readiness to address home delivery improved from the baseline to the follow-up (Figure 3). The community at baseline was at the stage of 3, “vague awareness,” showing that the community members felt that home delivery was a concern but had no immediate motivation to address it. After 15 months of implementation of stage-matched educational intervention, mothers and the week had implemented many tactics that targeted improvements among the dimensions of community readiness for the promotion of institutional delivery and institutional delivery use; the community had progressed to stage 5, “preparation,” showing that the local leaders had begun planning efforts to address home delivery and that the community offered better support. Institutional delivery use was also improved. The community readiness dimension with low anchored rating scores at baseline was also increased (Figure 4). The CRM dimensions of “Community knowledge of the issue” at baseline were anchored ratings of 5 which are relatively high and increased by two.

The baseline scores of anchored rating for “Community effort”, “Leadership”, “Community climate” and “resource allocation” were low, and except “resource allocation” each increased from a score of one to three. The largest absolute increase in dimension was observed at “Community efforts”, from three to six on the anchored rating scale. Whereas the dimension of “resource allocation” was not observed to have any change. Lastly, the “leadership” dimension score increased from three to four and the “Community climate” dimension score increased from three to five. The combination of collaborating with different local systems and village-based activism was key to achieving community relevance and reach. Community´s stage of readiness to address home delivery, at baseline in December 2019 and 15 months year follow-up in April 2021. Anchored community readiness rating scores by readiness dimension, at baseline in December 2019 and 15 months year follow-up in April 2021.

**Community readiness from comparison communities:** this program was given to communities with a low level of readiness at baseline assessment [7]. Among the kebeles with a low level of readiness, six kebeles were selected for the intervention and the control group. The readiness of the community was assessed from the comparison group to see the effect of the intervention on the level of readiness. The result showed that there was a significant improvement in the level of readiness in the intervention group as compared to the control group. The overall readiness of the intervention group before the intervention was at stage 3, “Vague awareness” has improved into stage 6, “initiation” after a 15-month stage matched intervention has been conducted. Whereas the control group´s overall level of readiness before the intervention was 3, “Vague awareness” did not show any change in readiness after a 15-month follow-up period (Table 2).

**Table 2:** the level of readiness score in the intervention and control group before and after the intervention Central Gondar Zone, Northwest Ethiopia, 2021

S.N	Dimension	Intervention group-average readiness level before intervention	Control group-average readiness level before intervention	Intervention group-average readiness level after intervention	Control group-average readiness level after intervention
1	Community knowledge	5.35	5.03	9.00	5.64
2	Community effort	3.93	2.99	6.75	4.25
3	Leadership	3.06	2.47	4.75	2.95
4	Attitude of community	3.29	2.83	6.63	2.94
5	Resource	3.29	2.73	4.28	2.44
6	Overall readiness	3.78	3.21	6.28	3.64

**Figure 5:**
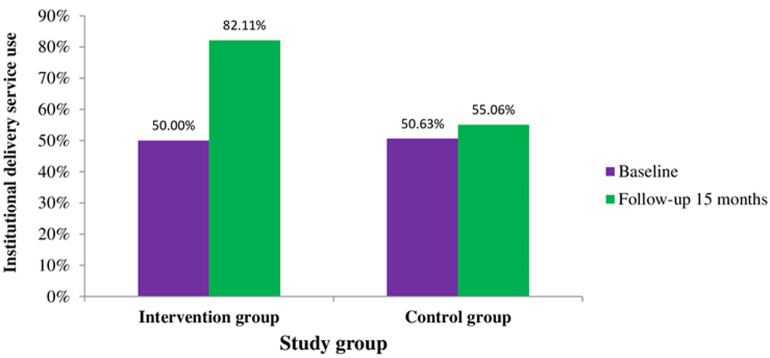
institutional delivery service use at the baseline and 15-month follow-up period in Central Gondar Zone, Northwest Ethiopia, 2021

The CRM dimensions of “community knowledge of the issue” for the intervention group at baseline were anchored ratings of 5 which are relatively high and increased by four after the 15-month intervention. This “community knowledge of the issue” in the control group at the baseline was anchored at a rating of 5, which remained unchanged after 15 months of follow-up. The baseline scores of the anchored ratings of the intervention group for “Community effort”, “Leadership”, “Community climate” and “resource allocation” showed an increasing readiness score. No significant change of dimension score was observed in the control group (Figure 5). The net effect of the intervention on the overall level of readiness was a score of two. The highest net effect of the intervention was observed on the attitude of the community and community knowledge of the issue, with a score of 3.22 and 3.04 respectively (Table 3).

**Table 3:** effect of the stage matched intervention on the readiness score of the community by dimension of the CRM, Central Gondar Zone, Northwest Ethiopia, 2021

Dimension	Readiness score of the intervention group after intervention (A)	Readiness score of the intervention group before intervention (B)	A-B= difference of intervention group (C)	Readiness score of the control group after intervention (D)	Readiness score of control group before intervention (E)	D-E= difference of control group (F)	C-F= net effect of the intervention on the readiness score (G)
Community knowledge	9.00	5.35	3.65	5.64	5.03	0.61	3.04
Community effort	6.75	3.93	2.82	4.25	2.99	1.26	1.56
Leadership	4.75	3.06	1.69	2.95	2.47	0.48	1.22
Community climate	6.63	3.29	3.34	2.94	2.83	0.11	3.22
Resource	4.28	3.29	0.99	2.44	2.73	-0.29	1.28
Over all readiness score	6.28	3.78	2.50	3.64	3.21	0.43	2.07

**Institutional delivery service utilization:** this study revealed that the prevalence of institutional delivery use at baseline was 49.797% (95% CI: 41.75-57.84%) and 50.513% (95% CI: 41.90-59.10%) in the intervention and control group respectively. After a 15-month follow-up period, it was 82.99% (95% CI: 65.40-100.60%) and 54.947% (95% CI: 45.60-64.30%) in the intervention and treatment groups respectively (Figure 6).

**Effect of the stage-matched educational intervention:** the difference-in-difference analysis showed that significant increases in overall community level of readiness (DiD=3.00; p<0.001) compared to the control group after 15 months of stage-matched educational intervention. The intervention group showed progressive (+3 score), whereas the control group did not change significantly. The scores of all the dimensions in the intervention group showed a significant increment. The Community climate score was shown to have a progressive score of +3 with statistical significance. In the DiD analysis, the dimension of community climate and resource allocation in the intervention group showed that an increment of a score of 3.00 (DiD=3.000; p<0.001) and 1.667 (DiD=1.667; p<0.001) respectively compared to the control group after a 15-month stage-matched intervention.

Similarly, community knowledge of the issue and community efforts in the intervention group showed an increment of a score of 3.333 (DiD=3.333; p<0.05) and 2,000 (DiD=2.000; p=0.094) respectively compared to the control group after 15 months of stage-matched educational intervention. On the other hand, the dimension of leadership in the intervention group showed an increment of a score by 1,000 (DiD=1.000; p=0.256) but not statically significant (Table 4). As noted earlier, we also tested the effect of stage-matched educational intervention on institutional delivery service use. Similarly, the effect of stage-matched educational intervention was significant for institutional delivery service use. Institutional delivery service use was increased by 28% (DiD=28.760; p<0.001) in the intervention group compared to the control group after 15 months of stage-matched educational intervention (Table 5).

**Table 4:** intervention versus control differences in community readiness scores at baseline and 15-months post-test: by community readiness dimension

Dimension	Before intervention (n=6 kebele)				After 15-month intervention (n=6 kebele)				Diff-in-Diff	
	C	I	Diff (I-C)	P-value	C	I	Diff (I-C)	P-value	DiD	P-value
Community knowledge	4.333	5.000	0.667	0.587	5	9	4	0.009***	3.333	0.081*
Community efforts	2.667	3.333	0.667	0.397	3.667	6.333	2.667	0.007***	2.000	0.094
Leadership	2.000	2.667	0.667	0.282	2.667	4.333	1.667	0.020*	1.000	0.256
Community climate	2.333	3.000	0.667	0.141	2.667	6.333	3.667	0.000***	3.000	0.001***
Resource	2.333	2.667	0.333	0.347	2.000	4.000	2.000	0.000***	1.667	0.008***
Overall community readiness	2.667	3.000	0.333	0.347	2.667	6.000	3.333	0.000***	3.000	0.000***

C: comparison area; I: intervention area; Diff: difference; DiD: difference in differences; *p< 0.05, **p< 0.01, ***p< 0.001

**Table 5:** effect of intervention on institutional delivery service use, Central Gondar Zone, Northwest Ethiopia, 2021

Indicators	Before intervention (n= 6 kebele)				After 15-month intervention (n=6 kebele)				Diff-in-Diff	
	C (%)	I (%)	Diff (I-C)	P-value	C (%)	I (%)	Diff. (I-C)	P-value	DiD (%)	P-value
Institutional delivery service utilization	50.513	49.797	-0.717	0.855	54.947	82.99	28.043	0.000***	28.760	0.001***

C: comparison area; I: intervention area; Diff: difference; DiD: difference in differences; *p< 0.05, **p< 0.01, ***p< 0.001

## Discussion

The current study adopted a stage-matched educational intervention based on the Community Readiness Model to improve the community level of readiness to promote institutional delivery use behavior in the rural community. This study indicated that the stage-matched educational intervention is an effective method for increasing the community level of readiness on the promotion of institutional delivery and increasing institutional delivery use behavior. This finding is in agreement with another study conducted for the promotion of exercise behavior [12]. This implies that a behavior change intervention needs to be based on the level of readiness at the baseline assessment of the targeted group because baseline information should be carried out in such a way that the same type of data can be collected after the intervention, in order to compare the results and assess the extent of change, or thereof [24]. After 15 months of intervention, in the current study, the community´s overall level of readiness for the promotion of institutional delivery and service use was significantly improved from baseline. At baseline, the overall level of community readiness to address home delivery was stage 3; this score was increased at the 15-month time point to stage 5. Similarly, institutional delivery use was increased from 50.4% to 66.9% at the 15-month time point. In contrast, the dimension of resource allocation did not have any changes. Previous studies support this finding [12]

These findings indicted that stage-matched educational intervention is more efficient in assisting the community to increase their readiness related to the promotion of institutional delivery, such as taking ownership, self-confidence to take action and behavioral strategies to be engaged in the institutional delivery service use [25]. The CRM measures (Community knowledge of the issue, community efforts, leadership, community climate, and resource allocation) differed significantly between the baseline and 15 months. The intervention activities carried out had inspired the participants to mobilize their respective communities at the village level by sharing successful experiences in changing the community's readiness and behavior for participation in promotional activities and service use behavior. Strategies influence barriers, knowledge, behavioral cues, and enhance community self-efficacy. This finding was supported by behavioral models for intervention at the community level and a systematic review and meta-analysis of effective behavior change [25-30]. To effectively change community health behavior, a multifaceted approach of assisting people to adopt, change, and maintain behavior was required. Maintaining a particular behavior over time might require different strategies than establishing that behavior in the first place. Models of behavior change help to guide strategies to promote healthy behaviors and facilitate effective adaptation to and coping with illness [31,32].

Pre-post community assessment using key informant interviews to assess community readiness on the specific health issue appeared to pick up significant or marginally significant effects where we believed they should have appeared, and did not find evidence of effects on dimensions where one would not expect impact given the intervention under study [33]. In the current study, dimensions are assessed indirectly through knowledgeable community informants, and we believe that community readiness pre-post assessments of interventions play an important role. This intervention was based at the community level [34]. Based on the key informant assessment result, the evidence for community-level impact suggests that the community-level intervention had at least some strength, in the level of readiness and service use. From our experience of the current study, we believe that the key informant community readiness assessments have a valuable role to play in evaluating non-randomized community trials, by providing formative insights into community dynamics, by providing a basis for matching community assignment to condition, by offering a tool that can be used directly with community activists in the stage-match training setting, and by providing a means of assessing community level of readiness and institutional delivery service use.

**Strengths and limitations:** as a prominent strength in applying CRM is the ability to analyze the various dimensions of CRM independently, this allows the formulation of plans to challenge each dimension in its own right. The CRM is used to determine the overall readiness of the community to prevent or promote a specific health issue. Whereas, as a limitation in applying the CRM method, emphasis on key informant views. This means that the community´s level of readiness could be understood at best as a proxy measure that bypasses the people who are most likely to be affected by the interventions-the residents of the target area. At the same time as the CRM handbook does assure the inclusion of community voices, it does not allow them to be included in determining the overall community readiness score. The method could be modified or augmented, broadening the definition of key respondents perhaps and using other qualitative methods to seek resident views on barriers and facilitators to their participation [35].

## Conclusion

The stage-matched educational intervention has a significant effect on the level of readiness and institutional delivery use. The community readiness model is an effective way to design an appropriate evidence-based behavior change intervention at the community level. Essential behavior change is required by focusing on community wide practice rather than individual behavior. The gap of effectiveness in the behavior change process could be bridged by the full engagement of the community via mandating and point of choice information. Providing support for individuals desiring to change is a dimension of the social practice approach, via placing historic and contemporary social and cultural forces at the center is a role of behavioral theory. Community-based social practices can be disrupted over time through the incremental interplay of community engagement. However, the circumstances and pace of disruption are rooted in understanding social and cultural causation, and how this underpins the distribution and acceptance of behaviors in a population.

### What is known about this topic

- *There is low level of community readiness for the promotion of institutional delivery service utilization;*
- *Stage-matched educational intervention has the potential to reinforce individual behavior change;*
- *Communities greatly vary in their interest and willingness to try new strategies and to be involved as a change agent*.

### What this study adds

- *Stage-matched behavior change remains limited in community-based intervention for the improvement of health service utilization, no studies had been there on the stage-matched behavior change intervention for the improvement of health service utilization in developing countries, particularly related to institutional delivery service;*
- *We used a stage-matched community-based health promotion intervention to provide behavior change of the community aimed at increasing the use of institutional delivery service;*
- *Our analysis and methodology serve as a model to other community-based behavior change intervention planning to perform impacts of stage-matched behavior change analysis*.
